# Felodipine Promotes the Recovery of Mice With Spinal Cord Injury by Activating Macrolipophagy Through the AMPK‐mTOR Pathway

**DOI:** 10.1111/jcmm.70543

**Published:** 2025-04-21

**Authors:** Yuqin Mao, Jinlong Wan, Binghao Lin, Pengtao Xu, Ke Zhang, Mengyun Jin, Shaoyan Xuan, Minxiu Wang, Jiqing Du, Lin Zhang, Zhihua Tang

**Affiliations:** ^1^ Department of Pharmacy Shaoxing People's Hospital Shaoxing China; ^2^ Department of Gastroenterology Gaozhou People's Hospital Maoming China; ^3^ Department of Orthopaedics The Second Affiliated Hospital and Yuying Children's Hospital of Wenzhou Medical University Wenzhou China; ^4^ School of Life and Health Technology Dongguan University of Technology Dongguan China

**Keywords:** AMPK, Felodipine, macrolipophagy, microenvironment, spinal cord injury

## Abstract

Spinal cord injury (SCI) is a serious clinical condition characterised by extensive mechanical damage that compromises the tissue structure and microenvironment of the affected area. This damage leads to the formation of fibrotic blood vessels and impaired energy metabolism, both of which hinder recovery. Felodipine, a clinically approved antihypertensive drug, acts as a selective calcium antagonist, primarily inhibiting extracellular calcium influx in arteriolar smooth muscle and selectively dilating arterioles. Additionally, felodipine has been demonstrated to induce autophagy. Considering these properties collectively, we hypothesised that felodipine could modulate the microenvironment of the injured spinal cord. In this study, we employed immunofluorescence and Western blot analyses to evaluate the effects of felodipine on microenvironment repair and neuroprotection, both in vitro and in vivo. Particular attention was given to its regulatory role in AMPK‐mTOR pathway‐mediated macrolipophagy. Our results demonstrated that felodipine effectively improved the injured spinal cord microenvironment by activating macrolipophagy, facilitating the clearance of myelin debris. Furthermore, felodipine promoted the restoration of endothelial cell tight junctions, thereby enhancing the integrity of the blood–spinal cord barrier. This attenuation of barrier disruption after SCI contributed to improved neuronal survival. These findings expanded the clinical application prospect of felodipine and presented new therapeutic avenues for treating SCI.

**Abbreviations**
AMPK: Adenosine 5′‐monophosphate (AMP)‐activated protein kinase
AMPK IN3: AMP‐activated protein kinase inhibitor 3
BSA: Bovine serum albumin
CD31: Platelet endothelial cell adhesion molecule‐1
DMEM: Dulbecco's modified Eagle's medium
DMSO: Dimethyl sulfoxide
FBS: Foetal bovine serum
HBMECs: Human brain microvascular endothelial cells
LC3: Light chain 3
MBP: Myelin basic protein
mTOR: Mammalian target of rapamycin
NeuN: Neuronal nuclei antigen
SCI: Spinal cord injury
VECs: Vascular endothelial cells
ZO1: Zonula occludens protein 1

## 1 Introduction

Spinal cord injury (SCI) is a serious central nervous system condition [1]. The severe destruction of tissue structure and the deterioration of the microenvironment following SCI result in vascular fibrosis and impaired energy metabolism, which significantly hinders recovery [2, 3].

The intricate vascular network within the spinal cord facilitates nutrient and gas exchange, which is critical in maintaining the region's microenvironments [4, 5]. As such, the protection and restoration of vascular function following SCI have increasingly become a focus of research. Mechanical damage caused by SCI triggers the release of large amounts of calcium ions, resulting in an overload within surviving cells [6]. Moreover, excessive calcium ions induce vasoconstriction in blood vessels, impeding blood transportation and essential nutrients. Furthermore, the accumulation of myelin debris and the development of vascular fibrosis following SCI are critical factors that should not be overlooked [7]. Zhou et al. reported that vascular endothelial cells (VECs) engulf myelin debris following SCI, which produces neutral lipids and vascular fibrosis [8]. As a result, identifying a pharmacological agent capable of antagonising calcium ions and modulating lipid metabolism has emerged as a key objective in efforts to regulate the microenvironment and improve recovery following SCI.

The antihypertensive drug felodipine is widely used in clinical practice. As a selective calcium antagonist, felodipine primarily inhibits the influx of extracellular calcium into arteriolar smooth muscle, resulting in the specific dilation of arterioles [9, 10]. Furthermore, Siddiqi et al. demonstrated that felodipine activates autophagy, providing neuroprotection in the central nervous system [11]. Autophagy is a tightly regulated cellular process in which damaged organelles and macromolecules are degraded by lysosomes under the control of autophagy‐related proteins [12]. This mechanism is crucial for maintaining cellular metabolic homeostasis [13]. In 2009, Singh et al. revealed that autophagy reduces intracellular lipid accumulation by directing lipid droplets to lysosomes for degradation through macrolipophagy [14]. However, the potential role of felodipine in modulating macrolipophagy to regulate the microenvironment of the injured spinal cord remains unexplored.

In our study, we validated the inhibitory effect of felodipine on vascular fibrosis. We investigated its neuroprotective potential following SCI, specifically focusing on elucidating the regulatory pathway of macrolipophagy modulated by felodipine.

## 2 Materials and Methods

### 2.1 Reagents and Antibodies

Dulbecco's Modified Eagle's Medium (DMEM) and foetal bovine serum (FBS) were obtained from Gibco (California, USA). Detailed information on primary antibodies is provided in the antibody list (Table S1). Secondary antibodies were obtained from Abcam (Cambridge, UK). Felodipine and Bodipy were bought from MCE (NJ, USA).

### 2.2 Cell Culture

Human brain microvascular endothelial cells (HBMECs) were obtained from iCell (iCell‐h070, Shanghai, China) and cultured in high‐glucose DMEM supplemented with 100 μg/mL streptomycin, 100 U/mL penicillin and 10% FBS in a 37°C incubator with 5% CO_2_.

### 2.3 CCK8 Test

The cell counting kit‐8 (CCK8) assay was used to measure relative cell viability based on the principle that a cell's reducing capacity reflects its metabolic activity and cell number. Various concentrations of felodipine were tested (control, 10, 25, 50, 75, 100, 125, 150, 175 and 200 nM) to determine the optimal dosage. Cells were treated with the drug in a 96‐well culture plate. After incubation, the medium was aspirated, and a fresh medium containing 10% CCK8 solution was added to each well, which was incubated for 1 h. Absorbance at 450 nm was measured using a multifunctional microplate reader, and statistical analysis was performed using Prism software.

### 2.4 Cell Stimulation

#### 2.4.1 For Immunofluorescence Staining

A total of 5 × 10^4^ cells were seeded onto cell slides. Once the cell confluence reached 50%, the slides were divided into four groups: control group (dimethyl sulfoxide, DMSO), sodium oleate group (200 μM sodium oleate, a classical lipid droplet inducer), felodipine group (200 μM sodium oleate and 100 nM felodipine), felodipine + AMP–activated protein kinase inhibitor 3 (AMPK IN3) group (200 μM sodium oleate, 100 nM felodipine and 107 nM AMPK IN3) and felodipine + DC‐LC3in‐D5 group (200 μM sodium oleate, 100 nM felodipine and 200 nM DC‐LC3in‐D5). The cells were subjected to a 24‐h incubation period before immunofluorescence staining.

#### 2.4.2 For Western Blot Analysis

The cells were divided into five groups when the cell confluence reached 80%: Control group (DMSO added), sodium oleate group (200 μM sodium oleate added), felodipine group (100 nM felodipine), felodipine + sodium oleate group (200 μM sodium oleate and 100 nM felodipine added) and felodipine + sodium oleate + AMPK IN3 group (200 μM sodium oleate, 100 nM felodipine and 107 nM AMPK IN3 added). The cells were subjected to a 24‐h incubation period before Western blot analysis.

### 2.5 Bodipy Staining

#### 2.5.1 Staining Solution Configuration

The Bodipy compound (1 mg) was dissolved in 382 μL of DMSO to prepare a 10 mM stock solution. Subsequently, the stock solution was diluted by a factor of 1000 to obtain a staining solution with a final concentration of 10 μM.

#### 2.5.2 Cell Staining

Adherent cells were cultured on sterile coverslips. A coverslip was removed from the medium, and the excess medium was aspirated. Then, 100 μL of the working solution was added, shaken gently to completely cover the cells and incubated for 30 min at room temperature. After being washed twice with medium for 5 min each, fluorescence microscopy was used for observation.

#### 2.5.3 Tissue Section Staining

Mouse spinal cord tissue sections were subjected to deparaffinisation, hydration and antigen retrieval at room temperature. Antigen retrieval solution was purchased from Beyotime Biotechnology Co., LTD (Quick Antigen Retrieval Solution for Frozen Sections, P0090). Then, the sections were washed twice in PBS for 5 min and twice in distilled water for 3 min. The sections were placed in a wet box with a drop of 3% hydrogen peroxide and incubated for 10 min at room temperature. They were washed again for 3 min × 3 times with PBS and for 3 min × 1 time with distilled water. Subsequently, 100 μM Bodipy staining solution was applied to the tissue and incubated for 30 min. The aspirated Bodipy staining solution was washed thrice with PBS for 5 min each time. Finally, the tissue sections were imaged and observed under a fluorescence microscope.

### 2.6 Mice Model of SCI

A cohort of 60 adult female C57BL/6 mice, weighing between 18 and 20 g, was procured from Hangzhou Enlighten the Truth Laboratory Animal Technology Co. Ltd. for this study. All procedures were conducted according to the Guide for the Care and Use of Laboratory Animals from the National Institutes of Health and were approved by the Animal Care and Use Committee of Shaoxing People's Hospital (approval number: 2023Z079). The C57BL/6 mice were anaesthetised with 1% sodium pentobarbital, with the death rate of sodium pentobarbital of 0%. The T9–T10 spinous processes and lamina were removed to expose the spinal cord. A contusion injury was induced at T9 by dropping a heavy object (10 g; the impact tip was a small cylinder with a diameter of 2 mm) from a height of 5 cm. Postoperatively, intraperitoneal injections of 0.9% cefazolin sodium were administered twice daily, and artificially assisted urination was performed every morning and evening [15].

### 2.7 Animal Drug Delivery

First, six mice were selected and divided into two groups on average: the SCI group (SCI modelling) and the rapamycin group (2 mg/kg rapamycin was given daily following SCI modelling). Tissue samples were collected for testing exactly 7 days postadministration. Subsequently, the remaining 54 mice were evenly divided into three groups: Sham group (only the lamina was removed without SCI modelling), SCI group (SCI modelling, intraperitoneally injected with corn oil containing 10% DMSO) and felodipine group (5 mg/kg of felodipine dissolved in corn oil with 10% DMSO was intraperitoneally injected daily following SCI modelling). Tissue samples were collected precisely 7 and 28 days postadministration for analysis.

### 2.8 Evaluation of Functional Recovery of SCI Mice

The mice were allowed to move freely on a platform, and their BMS scores were evaluated based on hind limb stepping and forelimb placement [16].

### 2.9 Haematoxylin and Eosin (H&E), Nissl Staining and Masson Staining

Longitudinal tissue sections from each experimental group were subjected to pathological analysis. The sections underwent fixation, paraffin embedding, sectioning, dewaxing and gradient alcohol rehydration. They were stained with H&E (Solarbio: G1120, Beijing, China), Nissl (Solarbio: G1430, Beijing, China) and Masson (Solarbio: G1346, Beijing, China) stains according to the protocols provided with the respective staining kits. Subsequently, the sections were dehydrated using a gradient ethanol series, cleared with xylene and sealed with neutral resin. Observations were made under an optical microscope, focusing on the junction between normal and damaged tissue areas. For each index, three mice were analysed, and one region from each mouse was selected for observation.

### 2.10 Immunofluorescence Staining

Longitudinal tissue sections were deparaffinised, rehydrated and subjected to antigen retrieval. Subsequently, the tissue was blocked with 5% bovine serum albumin for 30 min. Primary antibody incubation was conducted overnight at 4°C, followed by a 1 h incubation with the corresponding fluorescently labelled secondary antibody. The DAPI staining was used to visualise the nuclei. The observation field was focused on the junction between normal and damaged tissue areas. For each index, three mice were analysed, and one region from each mouse was selected for observation. Image acquisition was performed using a Nikon Ti–E&A1 plus microscope. Statistical plots were generated using Prism software (version 9; GraphPad, San Diego, CA).

### 2.11 Western Blot Analysis

The extracted tissue was stored at −80°C for Western blot analysis. Protein was extracted from tissue homogenate, and Western blotting was performed using a 12.5% gel. Briefly, the protein was electrophoretically transferred onto polyvinylidene fluoride membranes, which were then blocked with 5% skim milk for 2 h and incubated with primary antibodies overnight at 4°C. After washing thrice with TBST, the membranes were incubated with HRP at room temperature for 1 h. Signal intensity was detected using the ChemiDoc XRS + imaging system (Bio‐Rad). Band density was quantified using Image J software.

### 2.12 Statistical Analysis

The BMS scores were analysed using generalised linear mixed models. A two‐sided student's t‐test was used to compare the data from two groups. Analysis of variance and Turkey's post hoc analyses were used to compare the data from more than two groups. Statistical analysis was performed using Excel and GraphPad Prism software (version 9). Data are displayed as mean ± standard deviation (SD). *p* < 0.05 indicated a statistical difference.

## 3 Results

### 3.1 Autophagy Activation Could Accelerate Lipid Metabolism in VECs Following SCI

**FIGURE 1:**
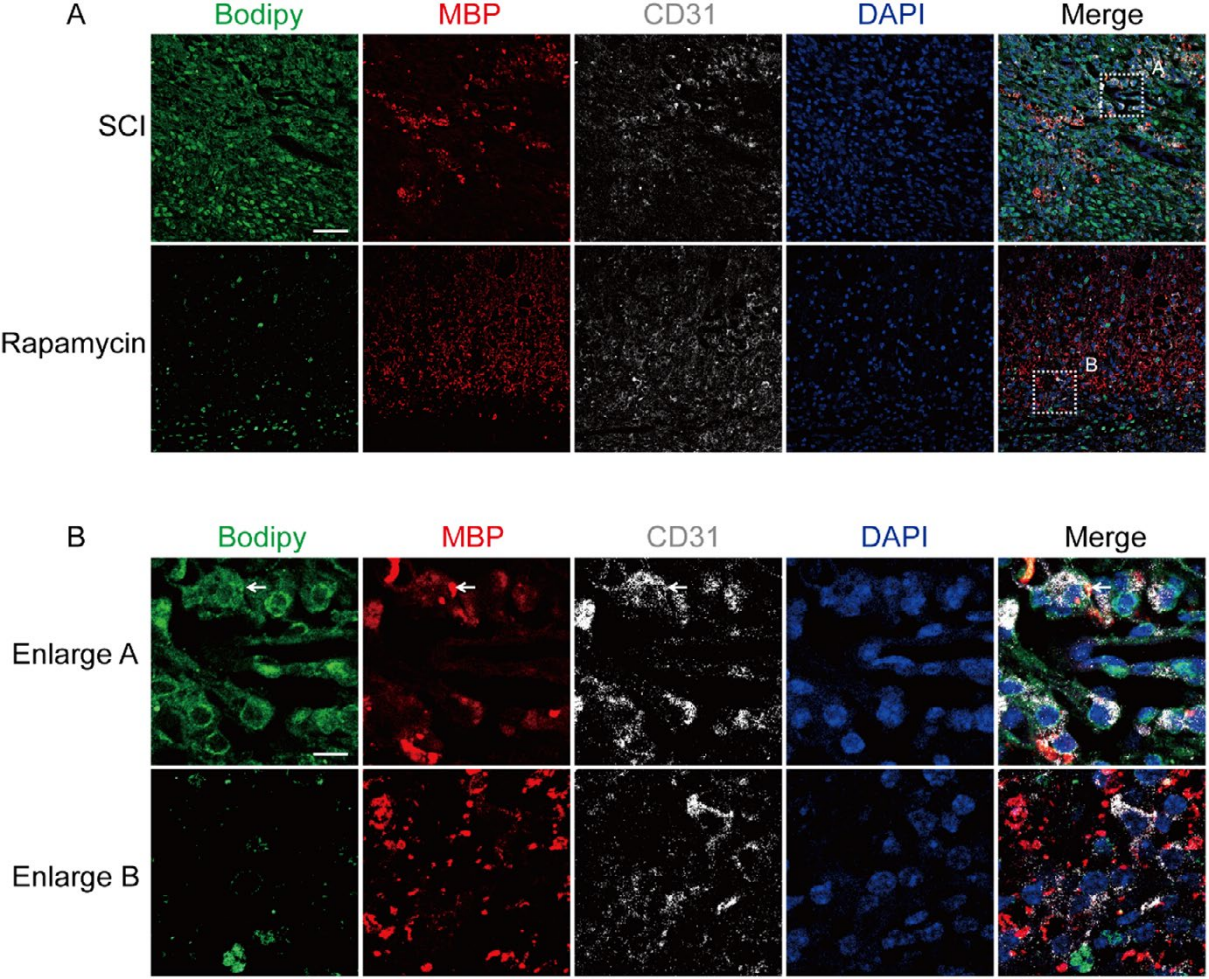
Autophagy activation could accelerate lipid metabolism in VECs following SCI. (A and B) Immunofluorescence staining of Bodipy (green), myelin basic protein (MBP) (red), CD31 (white) and DAPI (blue) in each group following SCI. The white boxes indicated the enlarged areas. The part conducted by the white arrow indicated the presence of large amounts of myelin debris (MBP) and lipids (Bodipy) in VECs (CD31). Magnification: ×20; scale bar: 100 μm.

Following SCI, a substantial amount of myelin debris was generated due to mechanical damage [17]. Extensive research has demonstrated that microglia could phagocytose this myelin debris, resulting in the production of neutral lipids [18, 19]. Zhou et al. also reported that endothelial cells phagocytosed myelin debris to produce neutral lipids, leading to vascular fibrosis [8]. In this study, we first examined the uptake of myelin debris and the production of lipids by endothelial cells following SCI using the immunofluorescence technique. The results indicated that myelin debris was present in endothelial cells following SCI and colocalised with lipid marker Bodipy (Figure 1A,B, enlarged A). Autophagy is an effective way to clear abnormal molecules and damaged organelles in cells. In 2009, Singh et al. revealed that lipophagy efficiently metabolises intracellular lipids. When rapamycin activated autophagy in mice, lipid accumulation in endothelial cells was reduced (Figure 1A,B, enlarged B). To further substantiate that rapamycin reduces lipid accumulation by activating autophagy, we conducted colocalisation staining experiments using LC3, CD34 and Bodipy. Our findings demonstrated that rapamycin significantly upregulated the expression of LC3 and markedly decreased lipid levels in vascular endothelial cells (Figure S1). In conclusion, the activation of autophagy can accelerate lipid metabolism in VECs due to phagocytosis of myelin debris.

### 3.2 Felodipine Could Attenuate Lipid Accumulation‐Induced Fibrosis in HBMECs by Inducing Autophagy Through the AMPK‐mTOR Pathway In Vitro

Following SCI, many cells undergo necrosis and apoptosis, releasing excessive calcium ions into the extracellular space. This calcium overload can lead to VEC spasms and impair their function [20]. Felodipine is a potent calcium antagonist widely used to dilate arterioles and alleviate hypertension [21] and has the potential to improve the vascular function of SCI. Besides, felodipine has demonstrated efficacy in inducing autophagy and safeguarding the central nervous system [11]. In this study, we focused on the role of autophagy in regulating lipid metabolism in endothelial cells and preventing endothelial fibrosis.

**FIGURE 2:**
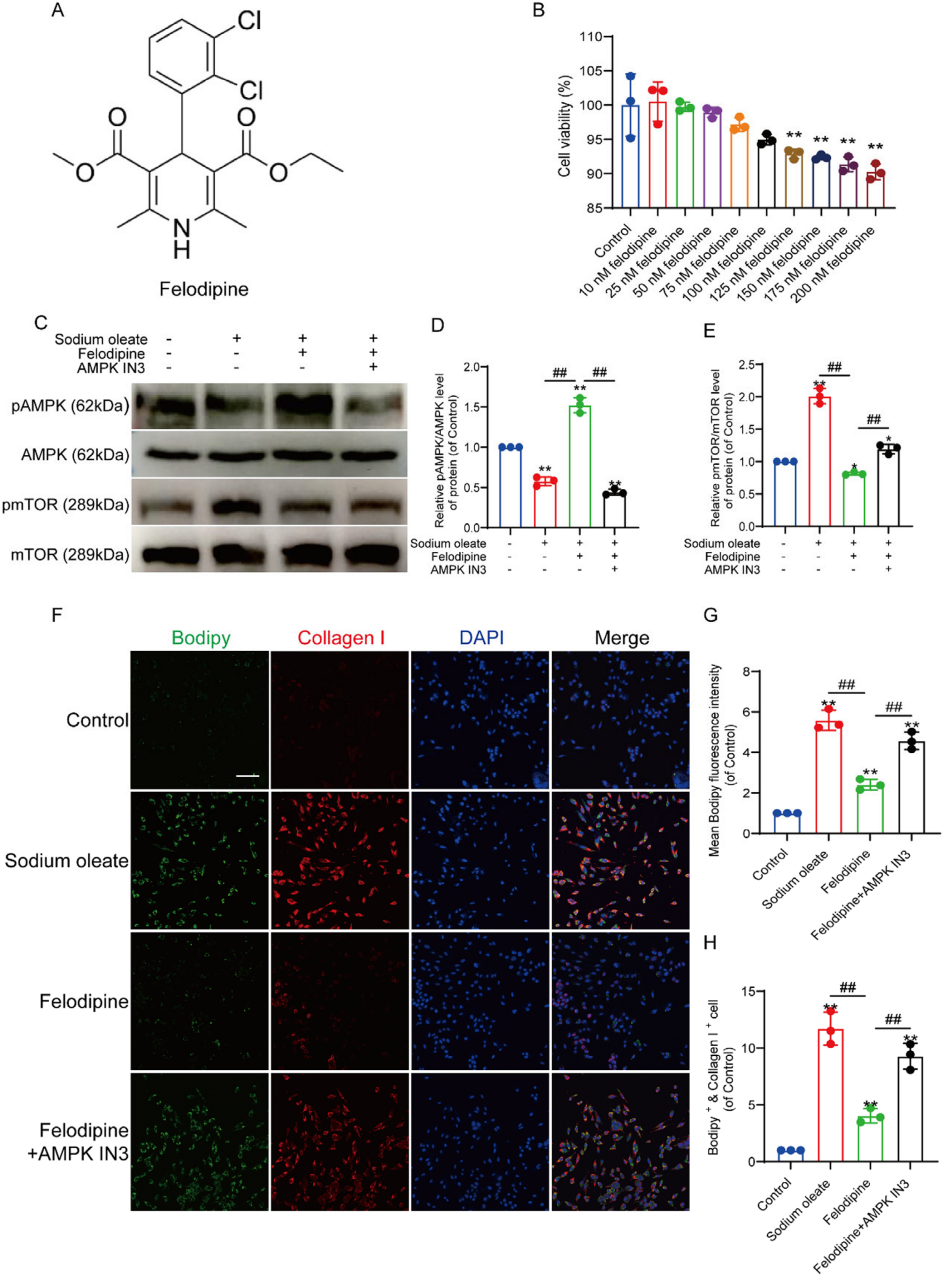
Felodipine could attenuate lipid accumulation‐induced fibrosis in HBMECs through the AMPK‐mTOR pathway in vitro. (A) The chemical structure formula of felodipine. (B) Adopting CCK‐8 to measure relative cell viability, the optimal concentration of felodipine was 100 nM in HBMECs. (C) Western blot indicates the expression of pAMPK, AMPK, pmTOR and mTOR in HBMECs. (D and E) Quantitative analysis of pAMPK/AMPK and pmTOR/mTOR protein expression. *Represents *p* < 0.05, **represents *p* < 0.01 versus the control group and ^##^represents *p* < 0.01. The data are the mean ± SD (*n* = 3). (F) Immunofluorescence staining of Bodipy (green), collagen I (red) and DAPI (blue) in HBMECs. Scale bar: 100 μm. (G) The mean fluorescence intensity of Bodipy in HBMECs. (H) Quantification of the proportion of Bodipy^+^ and collagen I^+^ cells.

**FIGURE 3:**
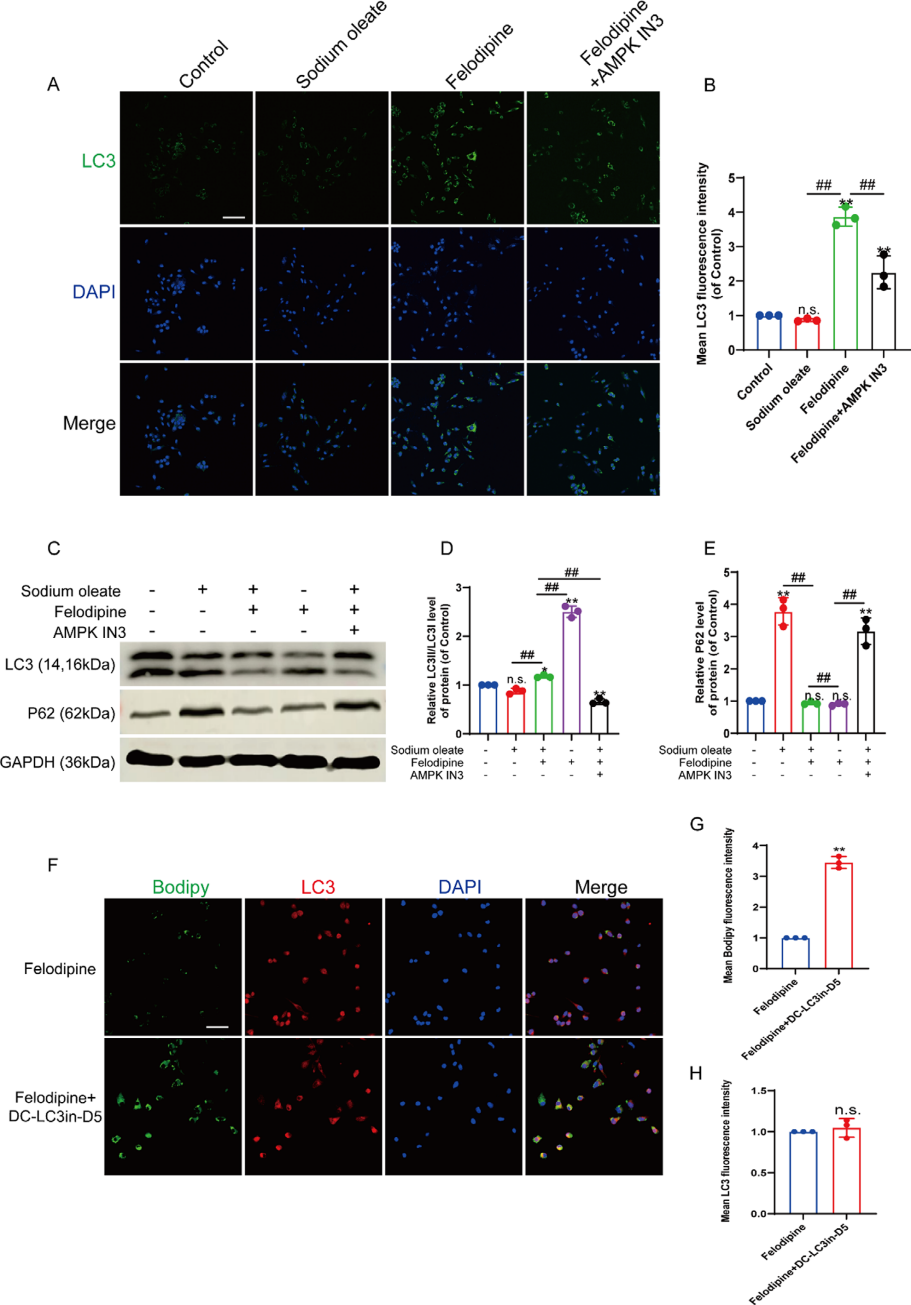
Felodipine could induce autophagy in HBMECs through the AMPK‐mTOR pathway in vitro. (A) Immunofluorescence staining of LC3 (green) and DAPI (blue) in HBMECs. Scale bar: 100 μm. (B) The mean fluorescence intensity of LC3 in HBMECs. (C) Western blot indicating the expression of LC3 and P62 in HBMECs. (D and E) Quantitative analysis of LC3II/LC3I and P62 protein expression. (F) Immunofluorescence staining of Bodipy (green) and LC3 (red) in HBMECs. Scale bar: 50 μm. (G and H) The mean fluorescence intensity of Bodipy and LC3 in HBMECs. N.S. (not significant), *represents *p* < 0.05, **represents *p* < 0.01 versus the control group and ^##^represents *p* < 0.01. The data are the mean ± SD (*n* = 3).

Additionally, due to the impaired vascular function and the disruption of energy metabolism homeostasis following SCI, we investigated the AMPK pathway, which plays a critical role in energy metabolism and autophagy [22, 23], so we made it the focus of our research. To determine the optimal concentration of felodipine, a CCK8 assay was conducted, and the results indicated that the optimal concentration of felodipine was 100 nM (Figure 2B). Western blot analysis demonstrated that the AMPK‐mTOR pathway of autophagy was inhibited by the induction of intracellular lipid accumulation by sodium oleate and reactivated by felodipine treatment. The expression of pAMPK was increased, and the expression of pmTOR decreased. Moreover, felodipine‐activated AMPK phosphorylation was suppressed by treatment with AMPK IN3, a specific AMPK inhibitor (Figure 2C–E). Western blot results from mouse spinal cord tissue similarly demonstrated that felodipine activated the AMPK‐mTOR pathway of autophagy after SCI (Figure S2). An immunofluorescence assay was performed to determine whether felodipine activation of the AMPK‐mTOR pathway could accelerate lipid metabolism and inhibit fibrosis in HBMECs. The results revealed that felodipine effectively inhibited lipid accumulation activated by sodium oleate in HBMECs and inhibited fibrosis. However, lipid accumulation and vascular fibrosis reemerged in HBMECs treated with AMPK IN3 inhibition (Figure 2F–H). Immunofluorescence and Western blot analysis were performed to determine whether felodipine promotes lipid metabolism through the autophagy pathway and suppresses vascular fibrosis. We examined autophagy markers LC3 and P62. The results demonstrated that felodipine effectively activated LC3, increased the expression of LC3II and accelerated the metabolism of autophagy substrate P62. Treatment with AMPK IN3 significantly inhibited autophagy (Figure 3A–E). To further verify that it was autophagy‐mediated lipid metabolism, we used the LC3 antagonist DC‐LC3in‐D5 to inhibit the autophagy process. The research data revealed that after the action of DC‐LC3in‐D5, although felodipine could still activate autophagy and increase the level of LC3, the lipid level in the cells did not decrease (Figure 3F–H). In conclusion, felodipine accelerates lipid metabolism and inhibits vascular fibrosis by activating autophagy through the AMPK‐mTOR pathway.

### 3.3 Felodipine Promoted Tissue Repair and Behavioural Recovery of Injured Spinal Cord

**FIGURE 4:**
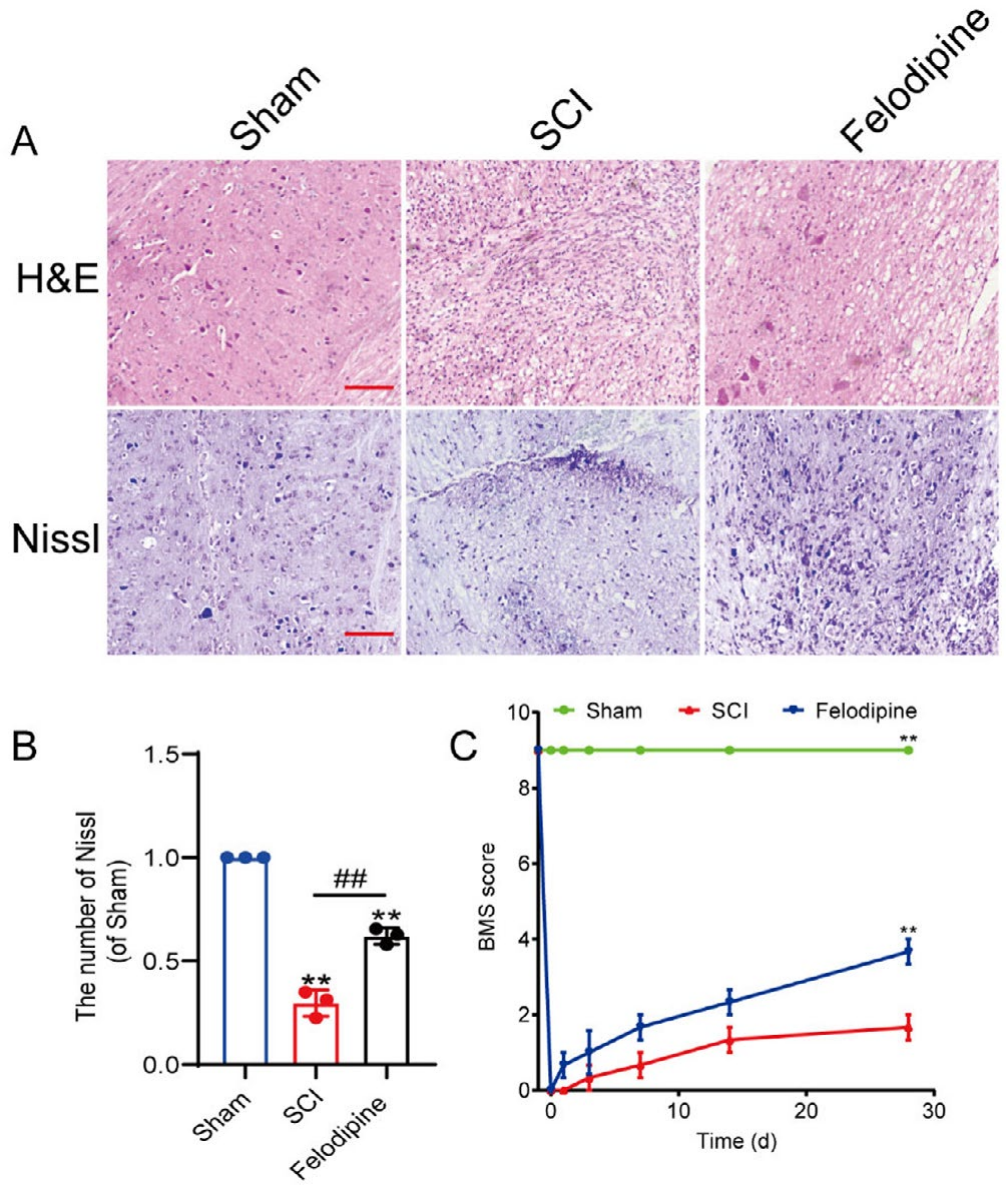
Felodipine promoted tissue repair and behavioural recovery of the injured spinal cord. (A) The H&E and Nissl staining spinal cord sections following SCI. Scale bar: 100 μm. (B) Counting analysis of Nissl staining positive neurons. (C) The BMS score of mice at the corresponding time points. **Represents *p* < 0.01 versus the sham group. ^##^Represents *p* < 0.01 versus the sham group.

Through histological and behavioural tests, we evaluated whether felodipine could promote recovery in SCI mice. The findings of H&E and Nissl staining indicated that the tissue structure of the mice was better repaired after felodipine treatment, with a noticeable degree of neuronal survival observed in the injured area (Figure 4A,B). Besides, BMS scores of mice indicated that their motor function improved following the felodipine treatment (Figure 4C).

### 3.4 Felodipine Could Inhibit VEC Fibrosis by Activating Autophagy In Vivo

**FIGURE 5:**
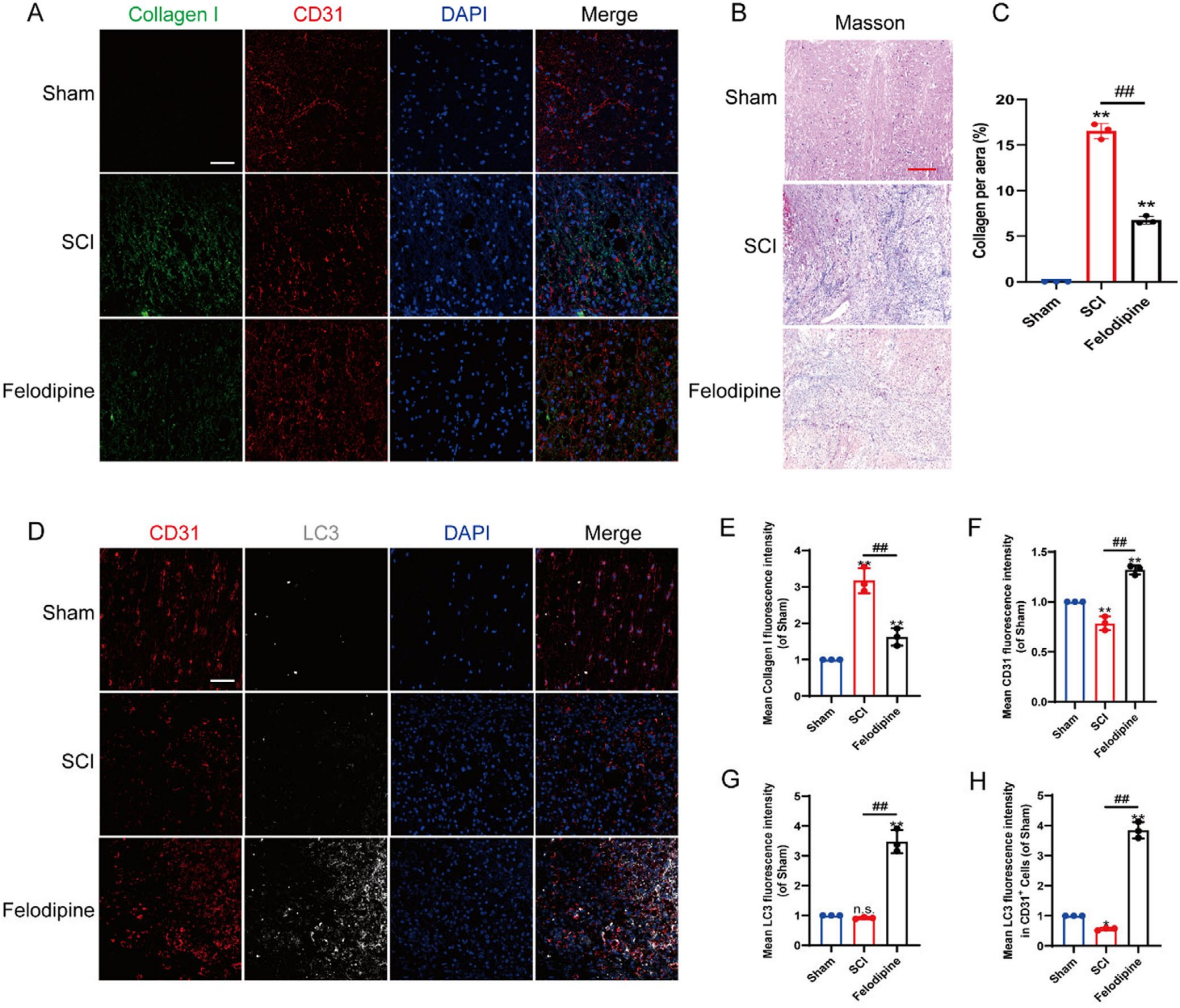
Felodipine could inhibit VEC fibrosis by activating autophagy in vivo. (A) Immunofluorescence staining of collagen I (green), CD31 (red) and DAPI (blue) in each group following SCI. Scale bar: 100 μm. (B) The Masson staining spinal cord sections following SCI. Scale bar: 100 μm. (C) Counting analysis collagen per aera by Masson staining. (D) Immunofluorescence staining of CD31 (red), LC3 (white) and DAPI (blue) in each group following SCI. Scale bar: 100 μm. (E and F) The mean fluorescence intensity of collagen I and CD31 in each group. (G and H) The mean fluorescence intensity of LC3 in each group. N.S. (not significant), **represents *p* < 0.01 versus the sham group and ^##^represents *p* < 0.01 versus the sham group. The data are the mean ± SD (*n* = 3).

To investigate whether felodipine could alleviate vascular fibrosis after SCI, we examined the decomposition of collagen I and CD31 using immunofluorescence. The results revealed that many fibrous substances were accumulated in the blood vessels following SCI, indicating the onset of vascular prevascularisation. Felodipine treatment effectively inhibited vascular fibrosis (Figure 5A,E,F). The results of Masson staining also revealed that a considerable amount of collagen deposition emerged after spinal cord injury (SCI), with the tissue morphology resembling that of blood vessels. After treatment with felodipine, the deposited collagen was markedly decreased (Figure 5B,C). To determine whether felodipine inhibits fibrosis through the activation of autophagy, we assessed the colocalisation of LC3 and CD31 by immunofluorescence. The results demonstrated that autophagy was significantly activated in endothelial cells after felodipine treatment (Figure 5D,G,H). In conclusion, felodipine inhibits vascular fibrosis by activating autophagy and improving the spinal cord microenvironment after SCI.

### 3.5 Felodipine Promoted the Restoration of Vascular Tight Junction and Protected Neurons In Vivo

**FIGURE 6:**
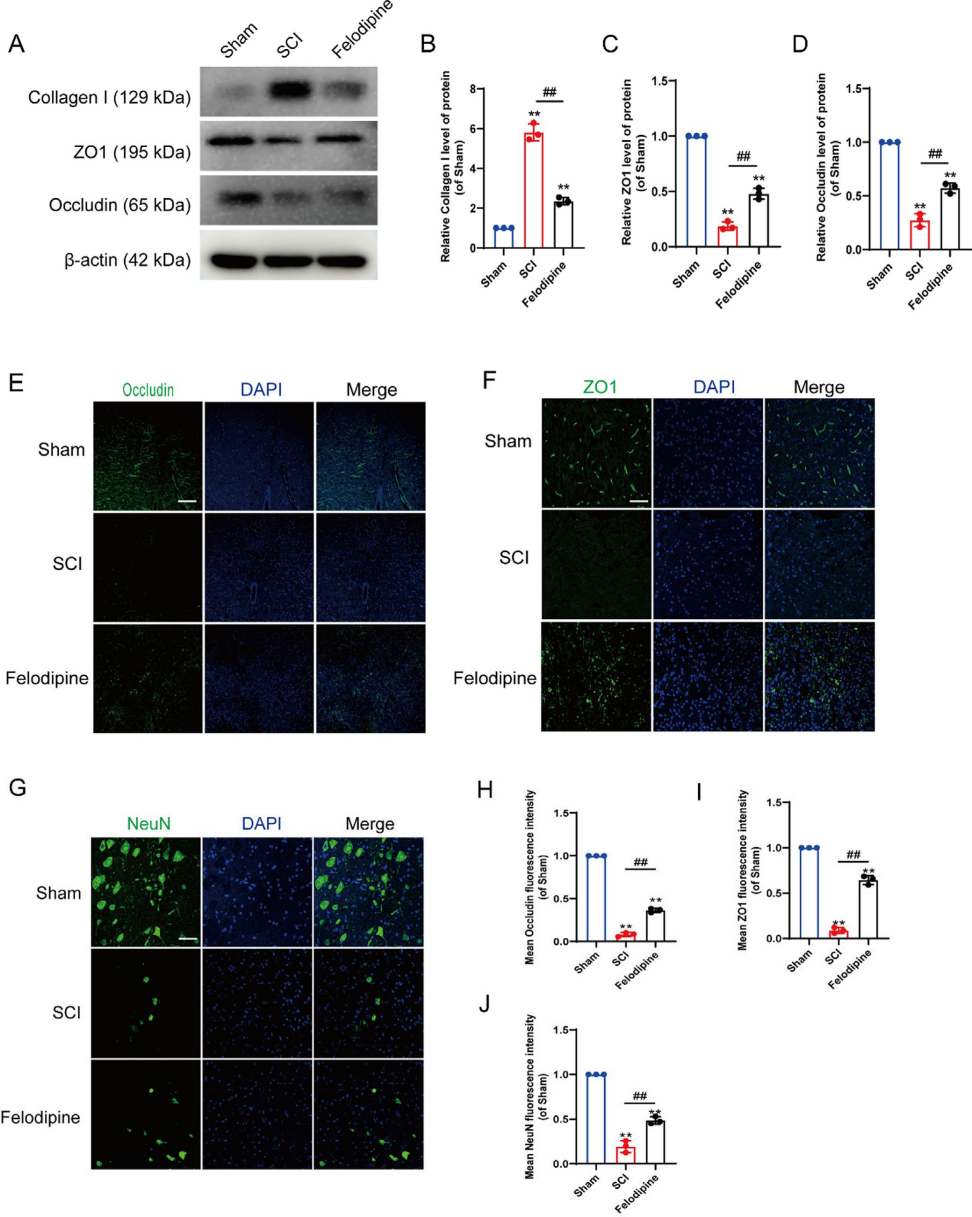
Felodipine promoted the restoration of vascular tight junction and protected neurons in vivo. (A) Western blot indicates the expression of collagen I, zonula occludens protein 1 (ZO1) and occludin in each group following SCI. (B‐D) Quantitative analysis of collagen I, ZO1 and occludin protein expression. (E) Immunofluorescence staining of occludin (green) and DAPI (blue) in each group following SCI. Scale bar: 100 μm. (F) Immunofluorescence staining of ZO1 (green) and DAPI (blue) in each group following SCI. Scale bar: 50 μm. (G) Immunofluorescence staining of Neuronal nuclei antigen (NeuN) (green) and DAPI (blue) in each group following SCI. Scale bar: 50 μm. (H–J) The mean fluorescence intensity of occludin, ZO1 and NeuN in each group. **Represents *p* < 0.01 versus the sham group. ^##^Represents *p* < 0.01 versus the sham group. The data are the mean ± SD (*n* = 3).

We subsequently evaluated many measures of vascular repair and neuronal protection. The relevant data of Western blotting (WB) demonstrated that felodipine could effectively safeguard the blood–spinal cord barrier and alleviate vascular fibrosis (Figure 6A–D). Immunofluorescence results also indicated that felodipine could effectively promote the recovery of tight junctions damaged by SCI (Figure 6E,F,H,I). Additionally, we tested the viability of neurons, and the research data indicated that felodipine could effectively promote neuron survival and significantly reduce the area of neuronal loss (Figure 6G,J and S3). Based on the analysis of the aforementioned results, felodipine ameliorates vascular fibrosis and effectively facilitates the restoration of the blood–spinal cord barrier. The stability of the vascular structure plays a vital role in regulating the microenvironment of the spinal cord and the survival of neurons.

## 4 Discussion

SCI is a severe clinical condition [24]. Previous research has primarily focused on neuronal protection [25]. However, increasing attention has recently been given to promoting SCI recovery by regulating the spinal cord microenvironment [26]. Following SCI, the destruction of blood vessels disrupts the energy supply, and the release of calcium ions due to cell death leads to calcium overload. This calcium overload contributes to the deterioration of the spinal cord microenvironment, triggering a cascade of secondary injuries [27, 28]. Consequently, regulating the spinal cord microenvironment after injury has become a significant area of research.

Following SCI, myelin fragments are phagocytosed and degraded into neutral lipids, causing lipid accumulation in cells of the central nervous system [29, 30]. This phenomenon is observed in phagocytic microglia and VECs, which engulf myelin fragments, degrade them into neutral lipids and induce vascular fibrosis [8]. This is a critical target for inhibiting the improvement of the injured spinal cord microenvironment. Consequently, enhancing the lipid metabolism of VECs is a crucial issue explored in this study. We observed that the spinal cord blood vessels can internalise myelin fragments and synthesise neutral lipids, with the metabolism of these lipids being intricately associated with autophagy.

Autophagy is a vital cellular mechanism that facilitates the removal of damaged organelles and foreign macromolecules [31]. Researchers have discovered that increased autophagy suppresses fat synthesis while promoting lipolysis [32]. This connection between autophagy and metabolism is called macrolipophagy [14, 33]. However, studies exploring the relationship between SCI and macrolipophagy are relatively limited. A recent study has found that myelin and nonmyelin debris contribute to the formation of foamy macrophages and lipid droplets following SCI^29^. In 2024, Ma et al. demonstrated that miR‐223 enhanced lipophagy by suppressing CTSB in microglia during lysolecithin‐induced demyelination in mice [34]. Similarly, Ou et al. reported that miR‐223 accelerates lipid droplet clearance in microglia following SCI by upregulating ABCA1 [35]. Despite these advancements, whether the activation of autophagy induces macrolipophagy to inhibit vascular fibrosis progression in the injured spinal cord remains uninvestigated. Consequently, we established a high‐lipid microenvironment in HBMECs using sodium oleate treatment. Lipid accumulation was assessed with Bodipy, and fibrosis was evaluated using collagen I. Our study demonstrated a positive correlation between increased lipid accumulation and aggravated vascular fibrosis. Furthermore, we observed that autophagy levels were inhibited in fibrotic vascular cells. These findings suggest that promoting macrolipophagy activation in vascular cells through an effective autophagy inducer could be a promising therapeutic strategy for SCI treatment.

Felodipine is a widely used antihypertensive drug. As a calcium antagonist, it effectively dilates small arteries and alleviates vasospasm [36]. Siddiqi et al. demonstrated that felodipine protects the central nervous system by activating autophagy [11]. These pharmacological properties make felodipine an ideal candidate for regulating the microenvironment of the injured spinal cord. In our study, we first confirmed that VECs engulf myelin after SCI, leading to significant lipid accumulation, which can be reduced by activating autophagy. Moreover, felodipine treatment inhibited HBMEC fibrosis by activating autophagy and reducing lipid accumulation in HBMECs. The energy metabolism of the injured spinal cord is blocked due to the disruption of the vascular supply. The AMPK‐mTOR pathway plays a crucial role in regulating energy metabolism in autophagy [37, 38]. The effects of felodipine on the activation of autophagy, the promotion of lipid metabolism and the inhibition of vascular fibrosis were significantly inhibited by inhibiting the AMPK‐mTOR pathway with the specific AMPK inhibitor AMPK IN3. These findings confirmed that felodipine activated macrolipophagy through the AMPK‐mTOR pathway. Furthermore, our in vivo experiments confirmed that vascular fibrosis mediated by lipid accumulation can influence the permeability of the blood–spinal cord barrier, resulting in the disorder of the spinal cord microenvironment and subsequently inducing neuronal death. Felodipine promotes SCI recovery by activating macrolipophagy, inhibiting VEC fibrosis and improving the microenvironment of the injured spinal cord. Undeniably, this study still has some limitations. Although we found that felodipine helped to promote the removal of myelin debris, the underlying mechanisms involving different cell types are not fully elucidated, such as microglia. Additionally, we recognise that this study lacks data from earlier time points post‐SCI, which limits our ability to fully understand the correlation between early behavioural improvements and molecular mechanisms. We did not use some widely accepted markers to define the lesion, such as glial fibrillary acidic protein (GFAP) to delineate the injury boundary or chondroitin sulphate proteoglycan (CSPG) to mark the injury centre. We plan to make these an important direction for future research to further understand the changes in the regional microenvironment of SCI.

**FIGURE 7:**
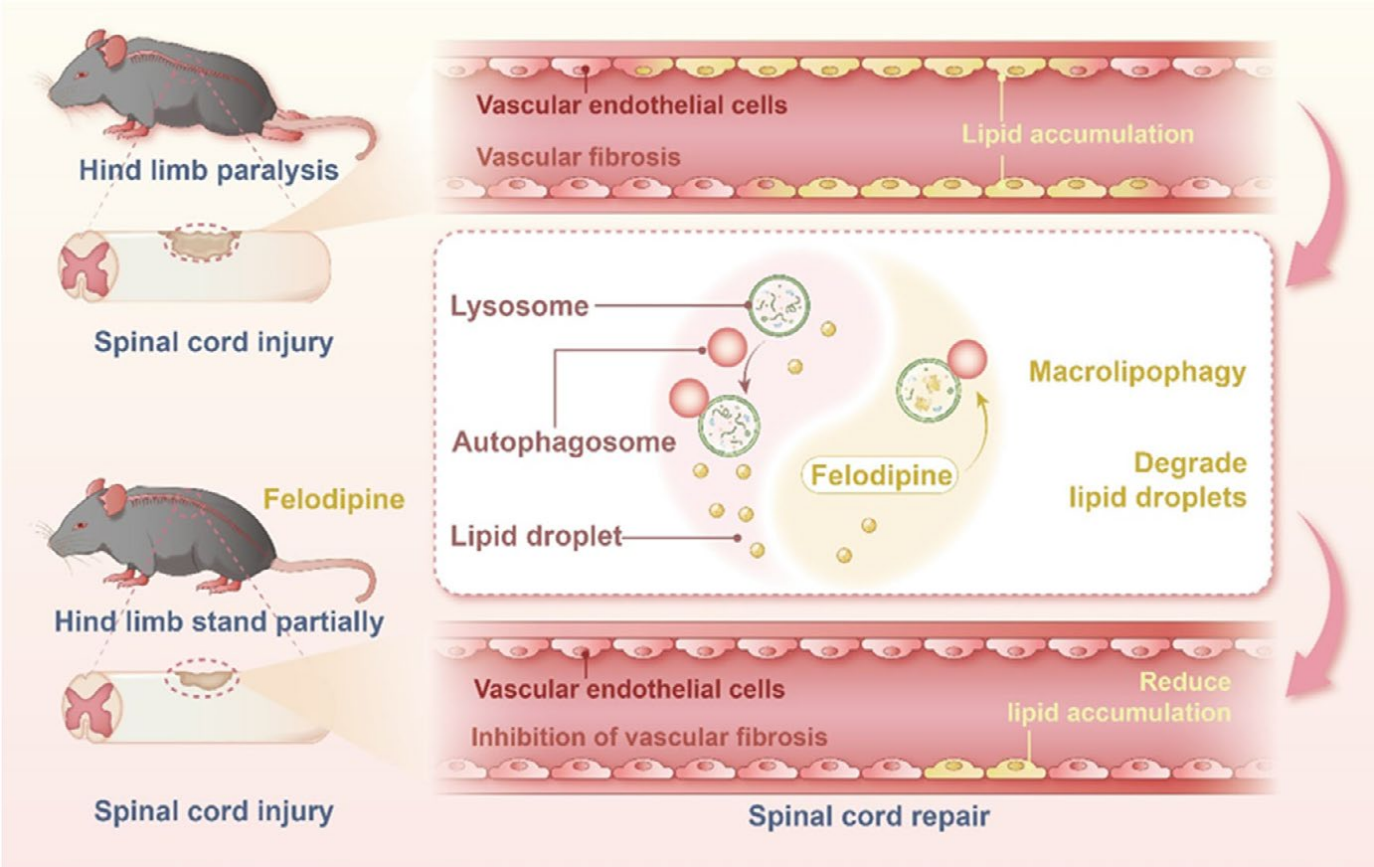
Felodipine promoted the recovery of mice with SCI by activating macrolipophagy. Schematic diagram indicating how felodipine promoted SCI recovery.

In conclusion, felodipine is an effective drug for modulating the SCI microenvironment (Figure 7). Our study has expanded the therapeutic scope of felodipine and revealed a new way for SCI repair.

## Author Contributions

**Zhihua Tang:** conceptualization (lead), data curation (equal), funding acquisition (lead), resources (lead). **Yuqin Mao:** conceptualization (equal), validation (lead), visualization (lead), writing – original draft (lead). **Jinlong Wan:** data curation (equal), visualization (equal), writing – original draft (equal). **Binghao Lin:** data curation (equal), validation (equal). **Pengtao Xu:** validation (equal). **Ke Zhang:** validation (equal). **Mengyun Jin:** validation (equal). **Shaoyan Xuan:** validation (equal). **Minxiu Wang:** validation (equal). **Jiqing Du:** data curation (equal), writing – review and editing (equal). **Lin Zhang:** data curation (equal), visualization (equal), writing – review and editing (equal).

## Ethics Statement

All procedures in this study were performed according to the Guide for the Care and Use of Laboratory Animals from the National Institutes of Health and approved by the Animal Care and Use Committee of Shaoxing People's Hospital (approval number: 2023Z079).

## Consent

The authors have nothing to report.

## Conflicts of Interest

The authors declare no conflicts of interest.

## Supporting information


Data S1.

## Data Availability

The data used to support the findings of this study are available from the corresponding author upon request.
