# Supplementary material for: Felodipine Promotes the Recovery of Mice With Spinal Cord Injury by Activating Macrolipophagy Through the AMPK‐mTOR Pathway
Source: J Cell Mol Med. 2025 Apr 21;29(8):e70543. doi: 10.1111/jcmm.70543 (PMC12011640; doi:10.1111/jcmm.70543)
Supplement: Supplementary file 1 — Data S1. [file JCMM-29-e70543-s001.docx]

**Table S1.** Antibodies used in the study

| **Antibodies** | **Company** | **Catalog** | **Application** |
| --- | --- | --- | --- |
| Anti-LC3 | Abcam | ab192890 | WB, IF |
| Anti-P62 | Abcam | ab109012 | WB |
| Anti-CD31 | Invitrogen | MA1-40074 | IF |
| Anti-CD34 | Affinity | AF5149 | IF |
| Anti-Collagen I | Abcam | ad260043 | IF |
| Anti-MBP | Invitrogen | MA1-24990 | IF |
| Anti-pAMPK | Invitrogen | 44-1150G | WB |
| Anti-AMPK | Invitrogen | AHO1332 | WB |
| Anti-pmTOR | CST | 5536S | WB |
| Anti-mTOR | CST | 2983S | WB |
| Anti-ZO1 | Affinity | AF5145 | IF |
| Anti-Occludin | Affinity | DF7504 | IF |
| Anti-NeuN | Abcam | ab104224 | IF |
| Anti-GAPDH | Zenbio | 380626 | WB |
| Anti-β-actin | Affinity | AF7018 | WB |

**
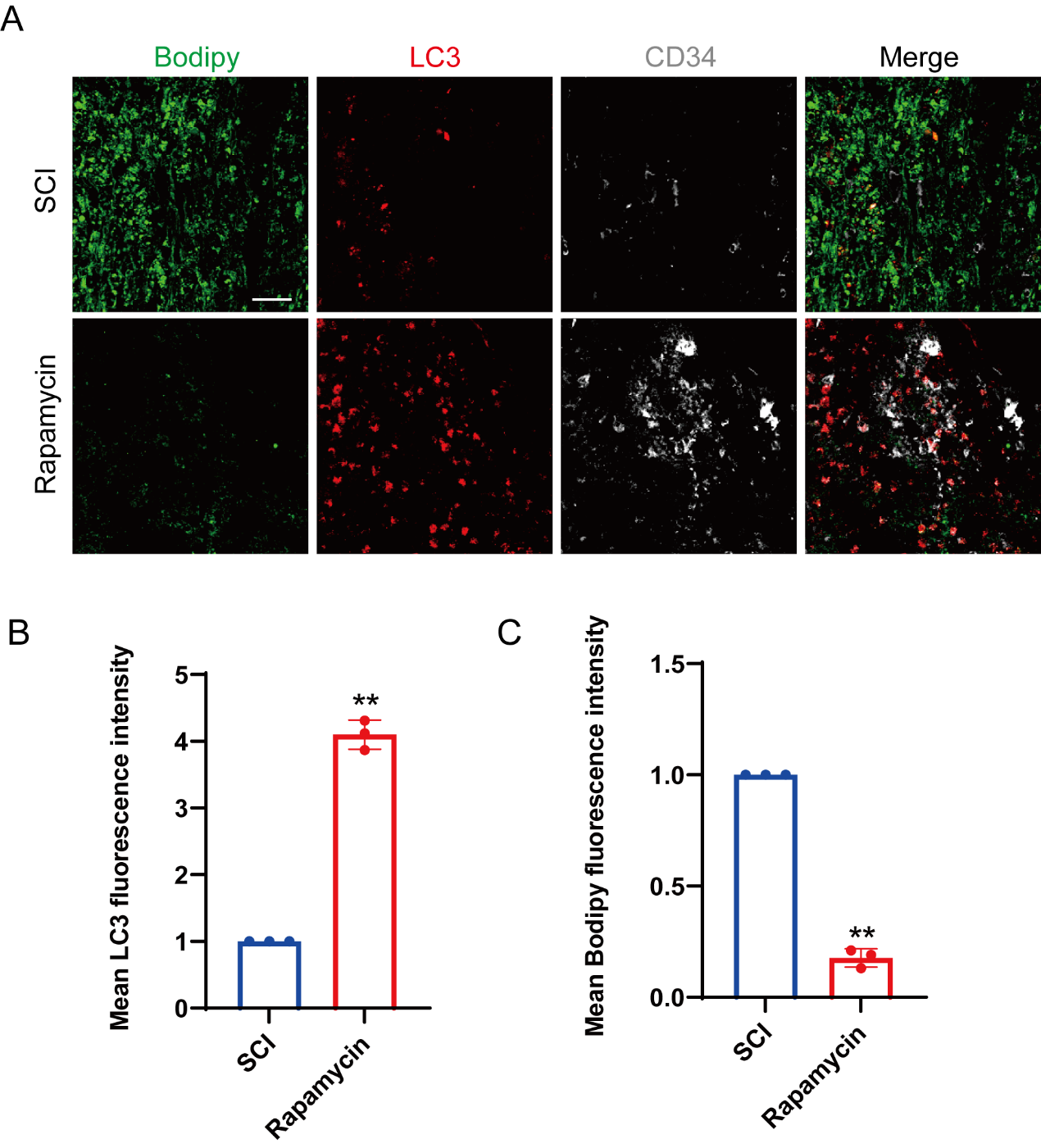
****Figure S1.** (A) Immunofluorescence staining of Bodipy (green), LC3 (red), CD34 (white) in each group following SCI. Magnification: × 20; scale bar: 100 μm. (B-C) The mean fluorescence intensity of Bodipy and LC3 in each group. **represents *p* < 0.01 versus the sham group. The data are the mean ± SD (n = 3).


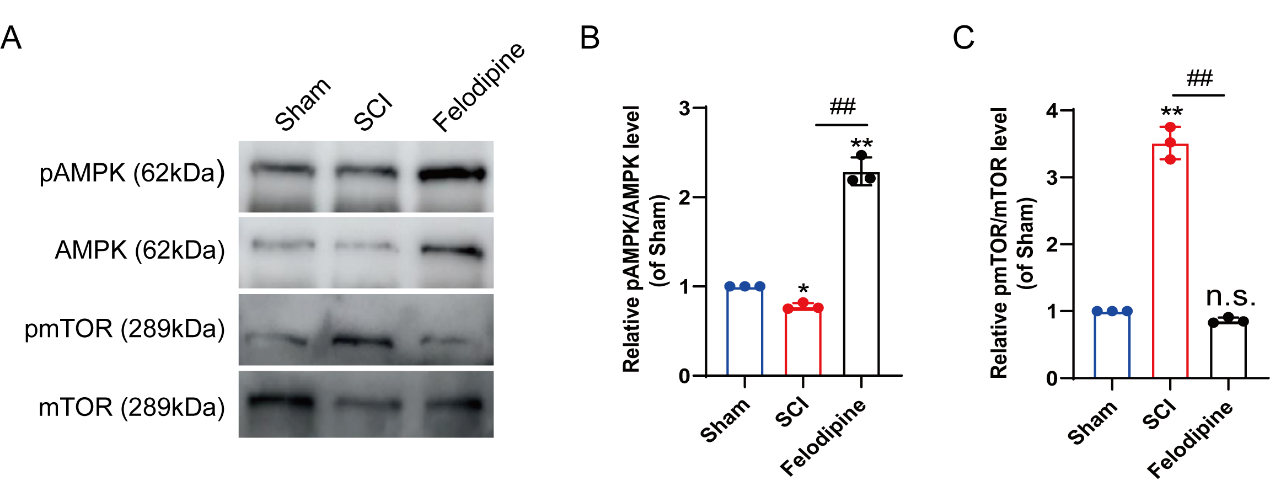


**Figure S2.** (A) Western blot indicates the expression of pAMPK, AMPK, pmTOR, and mTOR in each group following SCI. (B-C) Quantitative analysis of pAMPK/AMPK, pmTOR/mTOR protein expression. N.S. (not significant), *represents *p* < 0.05 versus the sham group. **represents *p* < 0.01 versus the sham group. ^##^represents *p* < 0.01 versus the sham group. The data are the mean ± SD (n = 3).

**
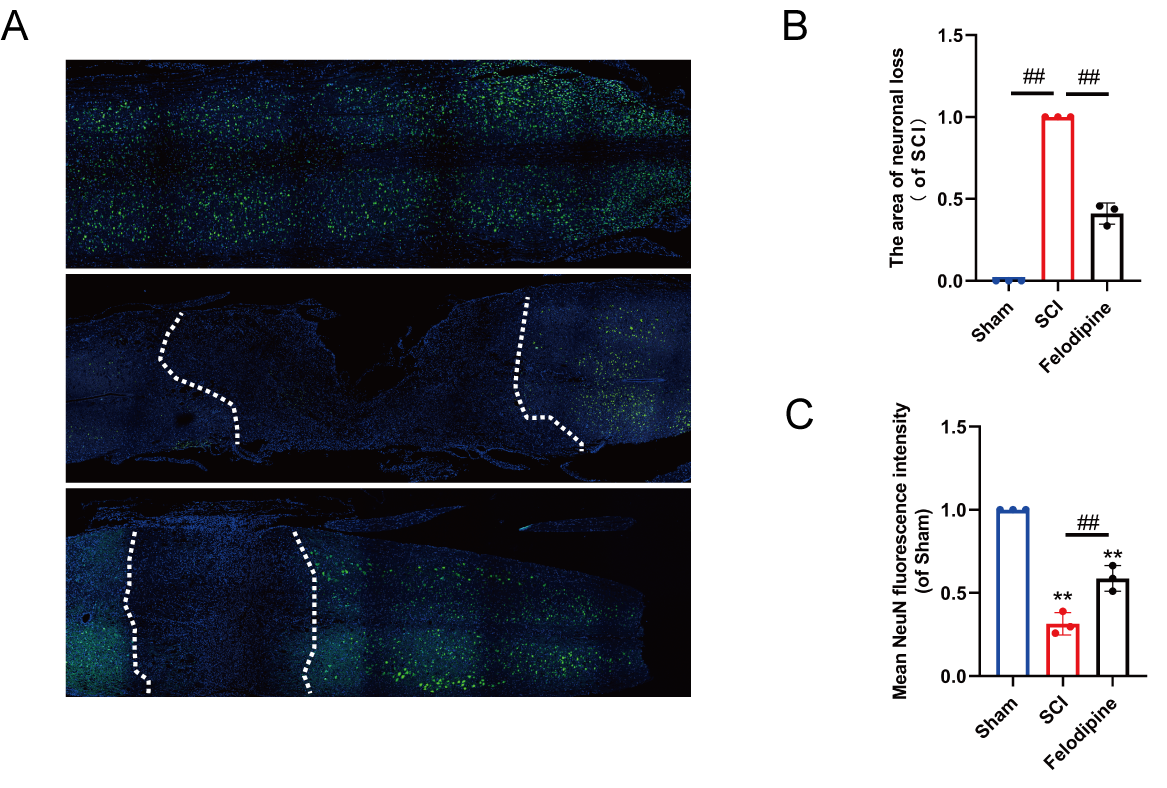
Figure S3.** (A) Representative immunofluorescence staining images of NeuN (green) and DAPI (blue) in spinal cord sections. (B) The area of neuronal loss in each group. (C) The mean fluorescence intensity of NeuN in each group. ^##^represents *p* < 0.01 versus the SCI group. **represents *p* < 0.01 versus the sham group. The data are the mean ± SD (n = 3).
